# Heterogeneous computing architecture for fast detection of SNP-SNP interactions

**DOI:** 10.1186/1471-2105-15-216

**Published:** 2014-06-25

**Authors:** Davor Sluga, Tomaz Curk, Blaz Zupan, Uros Lotric

**Affiliations:** 1 Faculty of Computer and Information Science, University of Ljubljana, Trzaska 25, SI 1000 Ljubljana, SI, Slovenia; 2 Department of Molecular and Human Genetics, Baylor College of Medicine, One Baylor Plaza, TX 77030 Houston, USA

**Keywords:** SNP-SNP interactions, Genome-wide association studies, Graphic processing unit, Many Integrated Core coprocessor, Intel Xeon Phi, CUDA

## Abstract

**Background:**

The extent of data in a typical genome-wide association study (GWAS) poses considerable computational challenges to software tools for gene-gene interaction discovery. Exhaustive evaluation of all interactions among hundreds of thousands to millions of single nucleotide polymorphisms (SNPs) may require weeks or even months of computation. Massively parallel hardware within a modern Graphic Processing Unit (GPU) and Many Integrated Core (MIC) coprocessors can shorten the run time considerably. While the utility of GPU-based implementations in bioinformatics has been well studied, MIC architecture has been introduced only recently and may provide a number of comparative advantages that have yet to be explored and tested.

**Results:**

We have developed a heterogeneous, GPU and Intel MIC-accelerated software module for SNP-SNP interaction discovery to replace the previously single-threaded computational core in the interactive web-based data exploration program SNPsyn. We report on differences between these two modern massively parallel architectures and their software environments. Their utility resulted in an order of magnitude shorter execution times when compared to the single-threaded CPU implementation. GPU implementation on a single Nvidia Tesla K20 runs twice as fast as that for the MIC architecture-based Xeon Phi P5110 coprocessor, but also requires considerably more programming effort.

**Conclusions:**

General purpose GPUs are a mature platform with large amounts of computing power capable of tackling inherently parallel problems, but can prove demanding for the programmer. On the other hand the new MIC architecture, albeit lacking in performance reduces the programming effort and makes it up with a more general architecture suitable for a wider range of problems.

## Background

We are witnessing a dramatic shift in the design of personal computer systems, where speedups are achieved by porting the parallel traits of supercomputers into the world of personal computing. Modern computers are heterogeneous platforms with many different types of computational units, including central processing units (CPUs), graphics processing units (GPUs), digital signal processors (DSPs), coprocessors and custom acceleration logic. Today’s CPUs contain from two to twelve cores, each capable of executing multiple instructions per clock cycle. Assisting the CPU, graphics processing units usually render 3D graphics, but can also provide a general-purpose computing platform. Current GPUs are designed as massively parallel processors offering substantially more computing power than CPUs. GPUs are the most powerful computational hardware available at an affordable price [1,2]. The availability of general-purpose GPUs with computing abilities in commodity laptop and desktop computers has generated a wide interest, including applications in bioinformatics [3-9].

The newest addition to the commodity computer parallel processing hardware is the Intel Xeon Phi family of coprocessors [10] designed for computationally intensive applications. Xeon Phi implements Intel’s Many Integrated Core (MIC) architecture and offers a theoretical performance similar to that of modern GPUs, but promises easier porting of existing software to the new architecture. Tianhe-2, currently the world’s fastest supercomputer has 48 000 Xeon Phi coprocessors [11].

Many computational problems in bioinformatics require substantial computational resources [12]. Problems that can be computed with a high degree of parallel and independent processing are most suited for heterogeneous massively parallel hardware. Our aim was to investigate how these modern architectures cope with problems that are typical for bioinformatics, such as the problem of SNP-SNP interaction detection. As a proof-of-concept, we focused on a parallel implementation of computational core for the web-application SNPsyn [13] by exploiting heterogeneous processing resources, multi-core CPUs, GPUs, and the new MIC coprocessors.

**Figure 1:**
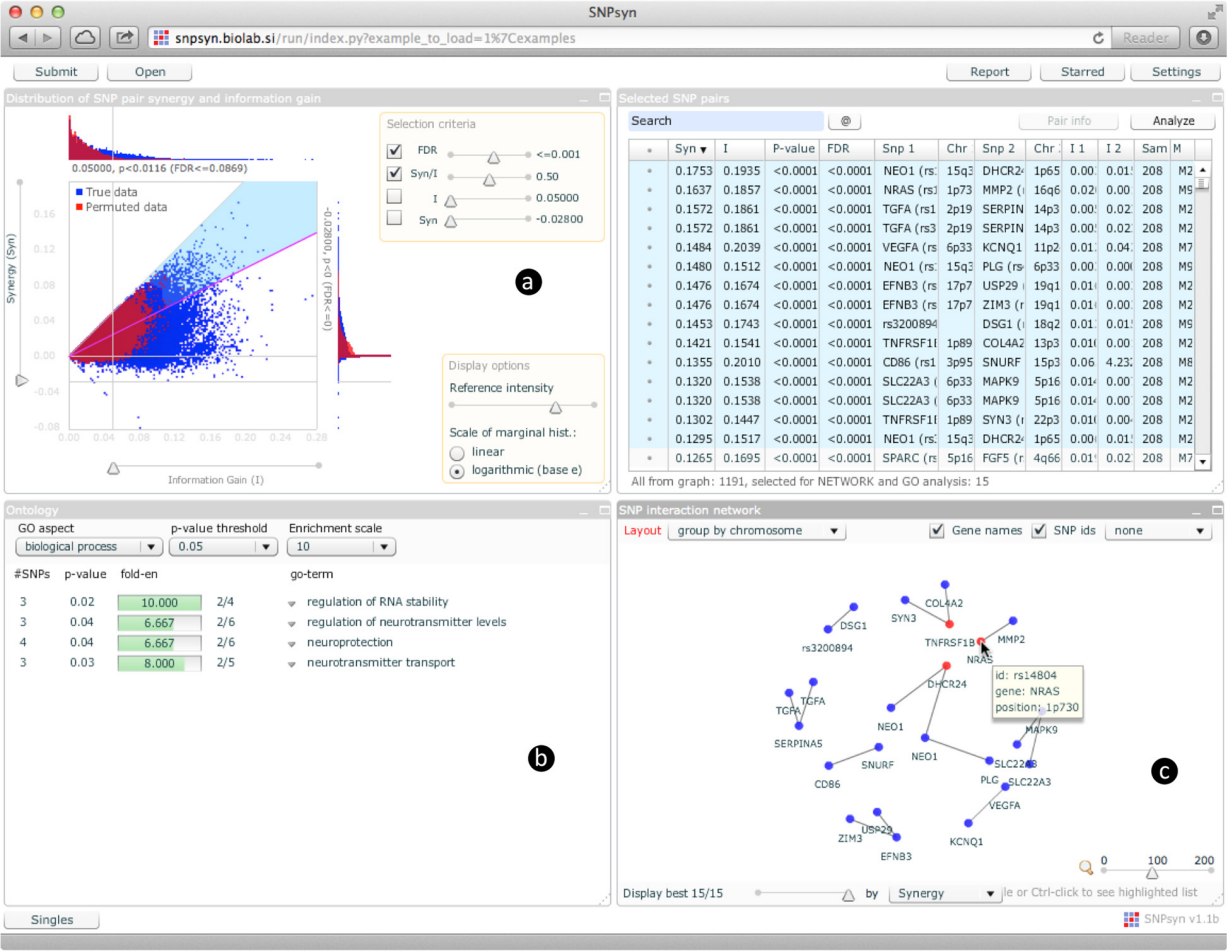
**SNPsyn graphical user interface.a)** A synergy versus information gain plot is used to select SNP-SNP pairs. **b)** Gene Ontology enrichment analysis for genes overlapping with selected SNP-SNP pairs. **c)** Synergy network of selected SNPs.

SNPsyn [13] (Figure 1) was developed as an interactive software tool for efficient exploration and discovery of interactions among single nucleotide polymorphisms (SNPs) in case-control genome-wide association study (GWAS) data. It uses an information-theoretic approach to evaluate SNP-SNP interactions [14]. Information gain is computed for every individual SNP, which allows the user to identify SNPs that are most associated with the disease under study. When searching for interesting pairs of SNPs, SNPsyn estimates the synergy between a pair of SNPs by computing the interaction gain. Information gain can identify SNP pairs with non-additive effects. Results are presented in an interactive graphical user interface that allows the user to select the most synergistic pairs, perform Gene Ontology enrichment analysis and visualize the synergy network among the selected SNP-SNP pairs.

SNPsyn computes the information gain exhaustively across all SNP pairs to avoid missing any pair where SNPs on their own provide no information about the phenotype under study. Because the number of pairs is quadratic to the number of SNPs, the exhaustive search quickly becomes computationally intractable for commodity computer systems. The information-theoretic-based detection of SNP-SNP interactions has a high degree of data parallelism and requires much more processing power than memory storage. This makes it a perfect candidate for processing on modern massively parallel architectures.

## Implementation

Below we describe the SNP-SNP interaction scoring approach we use in SNPsyn and discuss its implementation on CPU, CUDA and MIC architectures. Our particular concern is to evaluate Intel’s new MIC architecture and compare its advantages against currently prevailing CUDA architecture.

### SNP-SNP interaction scoring

**Figure 2:**
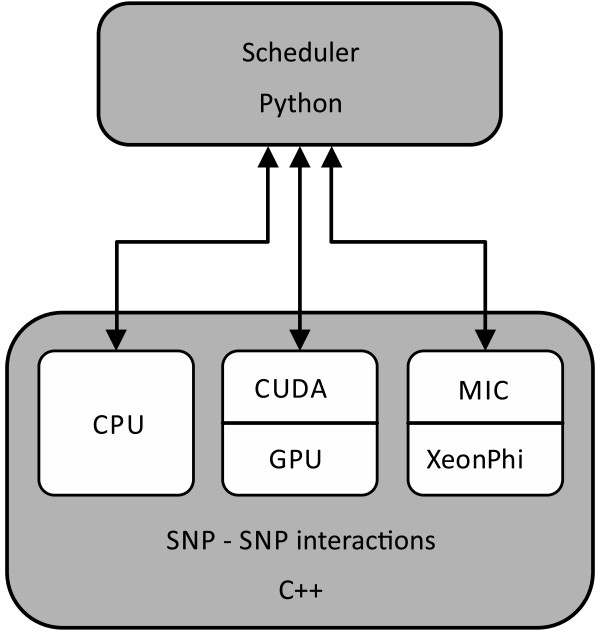
**SNPsyn software architecture.** Computation of SNP-SNP interaction is coded in C++ for the CPU, CUDA and MIC architectures. The scheduler that invokes the three heterogeneous implementations is written in Python.

The SNP-SNP interaction scoring scheduler, written in Python, partitions and distributes the computational tasks to all available, user-specified resources: CPUs, GPUs, and Xeon Phi coprocessors (Figure 2). It then merges the results from individual units into a final result file. Each thread (CPU, GPU or Xeon Phi) takes one pair of SNPs and performs all the calculations needed to compute the synergy score of the pair. The synergy of a pair of SNPs *X* and *Y* with respect to phenotype *P* is obtained by subtracting the information gains of individual SNPs from the information gain of the combined pair [13]:

(1) G(X,Y)=I(X,Y;P)-I(X;P)-I(Y;P).

Given the two SNPs and the phenotype as random variables *X*, *Y* and *P*, respectively, the information gains required in Equation 1 are calculated as [14]:

(2) $$\begin{array}{lcr} I(X;P) & = & \underset{x\in X,p\in P}{\sum }q(x,p)\underset{2}{\log}\frac{q(x,p)}{q(x)q(p)}, \end{array}$$

(3) $$\begin{array}{lcr} I(Y;P) & = & \underset{y\in Y,p\in P}{\sum }q(y,p)\underset{2}{\log}\frac{q(y,p)}{q(y)q(p)}, \end{array}$$

(4) $$\begin{array}{lcr} I(X,Y;P) & = & \underset{x\in X,y\in Y,p\in P}{\sum }q(x,y,p)\underset{2}{\log}\frac{q(x,y,p)}{q(x,y)q(p)}. \end{array}$$

Computation of marginal probabilities *q*(*x*), *q*(*y*), *q*(*p*) and joint probability distributions *q*(*x*,*p*), *q*(*y*,*p*), *q*(*x*,*y*), *q*(*x*,*y*,*p*) requires a single scan through case and control samples. The number of joint probability distributions *q*(*x*,*y*) and *q*(*x*,*y*,*p*) that need to be determined grows quadratically with the number of SNPs. This ensures enough computational load to compensate for the memory transfer costs and makes it efficient for an implementation on parallel hardware.

Permutation analysis is used to evaluate the significance of results on true data. Data is randomly shuffled thirty times. Each time, information gain and synergy for all pairs are calculated to obtain the null distribution, which is used to determine the significance of results on true data. Details on permutation analysis are described in Curk *et al.*[13].

### Parallel implementations of interaction scoring

Calculations are performed in parallel for as many pairs of SNPs as allowed by the hardware. We took special care to efficiently use the GPU and Xeon Phi hardware. We minimized memory transfers between the main CPU and the coprocessors to avoid bottlenecks and vectorized the code wherever possible. We optimized the number of threads running on the GPU to maximize throughput. To cope with the memory limitation of the GPU, SNPsyn includes optional heuristics to quickly estimate the importance of SNPs and reduce the data set prior to analysis. In the following sections we present the implementation details regarding both architectures.

### GPU and CUDA

GPUs gain their computational power from the numerous processing cores packed into one chip. For example, the modern Nvidia Tesla K20 GPU has 13 streaming multiprocessors, each containing 192 computational units called CUDA cores. These cores lack sophisticated control units and are thus likely to work best when executing the same instruction on many data elements in parallel with no divergent program paths in the algorithm. A programmer sees the GPU as a parallel coprocessor and can use it to speedup computationally intensive parts of the algorithm. Of course, there must be enough data parallelism in the code to make it worthwhile.

Different tools are available for programming GPUs. Nvidia offers the CUDA toolkit [15] for programming its own products. It includes a proprietary compiler and a set of libraries that extend the C++ syntax with parallel programming constructs. Another popular option is the OpenCL framework [16]. It supports hardware from different vendors but usually lags slightly in terms of performance when compared to specialized development kits such as CUDA.

**Figure 3:**
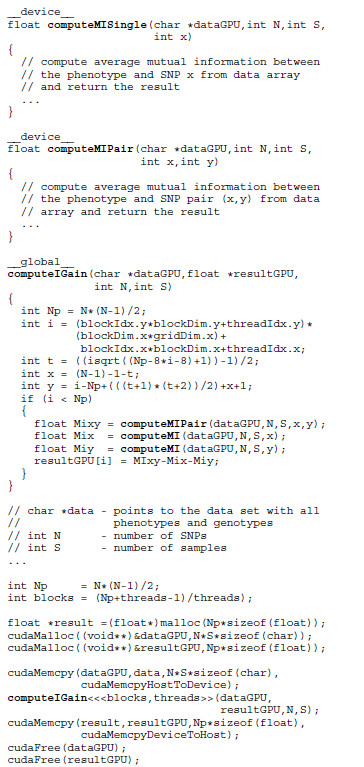
**CUDA code snippet.** Variables threads and blocks store the thread configuration. Function cudaMemcpy feeds the data into the GPU and retrieves the results afterwards. Each of the preconfigured GPU threads independently executes the **computeIGain** function and scores the associated SNP pair.

Regardless of the development tool used, the programmer must follow certain rules to obtain maximum performance [17]. The most important one is to partition the algorithm in blocks small enough to simultaneously start a sufficient number of threads to utilize all available resources. For example, consider the code snippet in Figure 3, a simplified version of a code that scores pairs of SNPs. Function computeIGain calculates the information gain of a SNP pair using Equation 1. The details of the calculation are omitted to emphasize the architecture specific parts of code. The snippet includes all the peculiarities of programming for GPUs. The program has to implement the GPU-specific part separately from the CPU code and explicitly transfer data from the host to the GPU. Special functions called kernels (marked with the keyword __global__) must be written to be executed on the GPU. Memory transfer and allocation functions must be called to supply the necessary data to the GPU and collect the results afterwards. Usually, the programmer performs measurements to determine which thread configuration is most suitable for a particular problem size and the appropriate number of threads to launch.

### Xeon Phi and MIC

Intel designed the Xeon Phi family of coprocessors around the new MIC architecture [18] to compete with GPUs specialized in general-purpose computing. The design follows a different approach in comparison to GPUs. Coprocessors consists of many simple, but fully functional processor cores derived from the Intel Pentium architecture. Intel improved the original design by adding a 512-bit wide vector unit and Hyper-Threading Technology. This enables Xeon Phi to achieve similar theoretical performance as modern GPUs. The model 5510P, which we used in this study, includes sixty cores interconnected with a bidirectional ring bus. Each core is capable of running four threads in parallel. The cores fetch data from the 8 GB of on-board RAM and communicate with the host CPU through the PCIe bus. In comparison to GPUs, each core on a Xeon Phi can efficiently execute the code even if threads do not follow the same program path. This makes it suitable for a wider range of problems, including multiplications of sparse matrices [19], and operations on trees and graphs [20].

**Figure 4:**
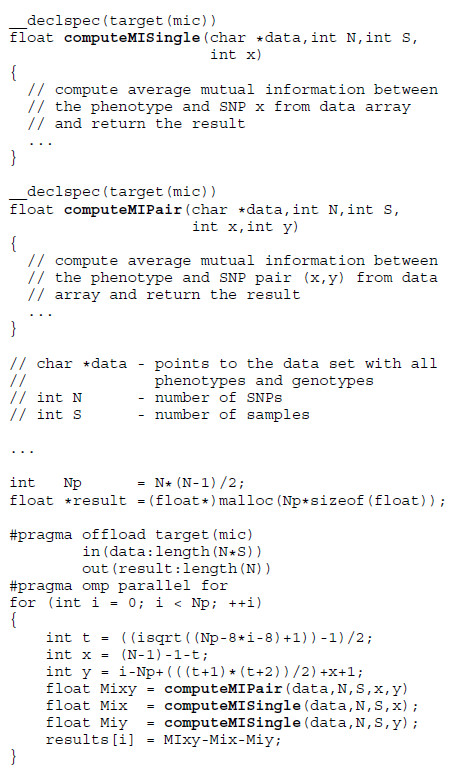
**MIC code snippet.** The first pragma directive marks the start of a MIC code section. Keywords in and out indicate the data to be transferred to and from the Xeon Phi. The OpenMP clause omp parallel for launches all available threads in parallel, which execute the code in the body of the loop and score the SNP pairs.

Intel provides a C++ compiler suite and all the tools needed to exploit the hardware [21]. The code can be parallelized using OpenMP directives or the MPI library and compiled for the MIC architecture. Resulting applications can then run only on the Xeon Phi coprocessors. Another, more general way to specify parallel execution is to use offload constructs along with OpenMP to mark the data and the code to be transferred and executed on the Xeon Phi. All other parts of the program will run normally on the host computer CPU. A third possibility is to use OpenCL framework in the same manner as with GPUs.MIC development tools facilitate data management through compiler directives. The example in Figure 4 demonstrates this programming paradigm. It performs the same operation as the snippet from Figure 3. The programmer marks the data and the code that is needed on the coprocessor. All memory allocations and transfers are done implicitly. To obtain best performance, the programmer must tailor the algorithms to fully utilize the vector unit. The Intel compiler automatically vectorizes sections of code where possible.

If a computer lacks Xeon Phi, the MIC code can be executed by the main CPU, which is not the case with CUDA-specific implementation. The MIC code looks much cleaner and easier to handle than CUDA code. The current drawbacks of using Xeon Phi are the shortage of supporting Linux distributions (officially only RedHat and SuSE) and the pricey development environment for the Windows operating system. The main aspects (relevant to the developer) of each of the architectures are shown in Table 1.

**Table 1:** Comparison of parallel computer architecture platforms with key aspects from the viewpoint of software development

	**x86/x64 single CPU**	**Nvidia GPU**	**Intel Xeon Phi**
**Tools**	Arbitrary compiler	CUDA Toolkit or OpenCL framework	Intel compiler suite
**OS support**	Many	Windows, Linux, Mac OSX	Linux (RedHat and SuSE), Windows
**Required programming skills**	Low	High	Medium
Lines of code*	260	460	360
**Programming remarks**	None	Architecture specific optimizations	Recommended optimizations using
		are crucial	vector unit
**Platform maturity**	Mature	Extensive documentation, many	Bugs in drivers, documentation needs
		programming examples	to improve

Lines of code (*) reports on the approximate length of the code that implements the computationally intensive tasks of SNPsyn.

## Results

We benchmarked SNPsyn on a workstation with two six-core Intel Xeon E5-2620 2.00 GHz CPUs capable of running up to twenty-four threads in parallel, 64 GB of RAM, two Nvidia Tesla K20 general-purpose computing cards with 5 GB of RAM each and one Intel Xeon Phi 5110P coprocessor with 8 GB of RAM. The operating system was CentOS 6.4.

**Figure 5:**
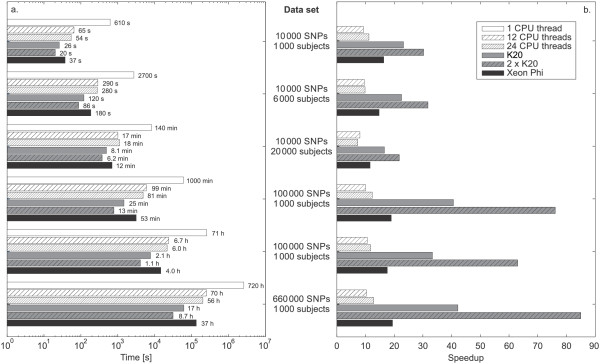
**Execution times and speedups achieved on various computing resources.** Shown are execution times on each hardware configuration for different problem sizes **(a)** and speedups in comparison to a single CPU thread execution **(b)**.

We evaluated the performance on a series of representative WGAS data sets constructed from the Infinium_20060727fs1_gt_MS_GCf data set found in the WTCCC study [22]. Our goal was to observe the effect of the number of SNPs and WGAS study subjects to the execution time on different configurations. We sampled with replacement the original data on 994 subjects and 15 436 SNPs to obtain data sets with the desired number of subjects and SNPS. We performed the analysis on data with 1 000, 6 000, and 20 000 subjects and 10 000, 100 000, and 660 000 SNPs. The study considered only the data sets that could fit into the GPU memory. Xeon Phi is clearly in advantage when compared to K20 regarding the amount of RAM (8 GB versus 5 GB). We tested six hardware configurations including one CPU core running a single thread, twelve CPU cores running twelve threads, twelve CPU cores running twenty-four threads, one GPU core, both GPU cores, and Xeon Phi.Figure 5 reports on execution times of the exhaustive SNP-SNP interaction analysis and the speedups achieved using various hardware configurations. For easier comparison, execution times are plotted on a logarithmic scale. As expected, execution times increase proportionally with the number of subjects and are quadratic with the number of SNPs included in the analysis.

The single thread CPU configuration takes more than 30 days to analyze the data on 660 000 SNPs and 1 000 subjects. Running twelve threads in parallel, one on each of the CPU cores, speeds up the computation by a factor of 10 and reduces the execution time to approximately 3 days. Increasing the number of threads to twenty-four reduces the time to perform the analysis to around 2 days with the speedup peaking at 12.8 compared to a one thread configuration. Memory bottleneck is the main factor for the poor speedup, which is far below the theoretical value of 24. Interestingly, similar speedups are achieved on all (smaller) data sets, meaning that there is enough data parallelism to keep the CPU busy.

Nvidia K20 provides for considerable reduction in execution times, with the analysis of the largest data set taking only around 17 hours, demonstrating a speedup of 42 in comparison to a single CPU thread. Sharing the work between both GPU cards doubles the speedup and reduces the execution time to 8 hours. Increasing the number of subjects leads to a noticeable decrease in speedup, as more data is being transferred between the main memory and the GPU. On the other hand, increasing the number of SNPs introduces more data parallelism into the computations, reflecting in an improved speedup.

Xeon Phi is positioned somewhere in-between K20 and CPU-only implementation. It achieves a speedup of nearly 20 on the largest data set, making the analysis run a day and a half, which is double the time needed on a K20. The speedup behaves similarly for Xeon Phi as for K20 – it increases with the number of SNPs and decreases with the number of subjects. This confirms that the drop is caused by transferring larger amounts of data without introducing additional parallelism.

Using only CPUs to analyze the data is unfeasible except for small data sets since the computations can take days to complete even on multiple cores. Xeon Phi provides a considerable performance boost with a maximum speed-up of nearly 20 and lots of on-board memory to store the data. Nvidia K20 clearly outperforms every other configuration in terms of speed and is the perfect choice when one wants to cut on the execution times as much as possible. This comes at a price of cumbersome programming and less on-board memory, which limits the size of data.

Technical specifications presented in Table 2 show similar trends: Nvidia K20 offers the highest theoretical performance in terms of TFLOPS and has the most complex design. Xeon Phi has considerably less computing power, but interestingly draws the same amount of power as K20 at maximum load. The Xeon E5-2620 CPU is the least efficient of all and lacks the performance to remain competitive at computationally intensive tasks.

**Table 2:** Technical specification of hardware platforms

	**Intel Xeon E5-2620**	**Nvidia Tesla K20**	**Intel Xeon Phi 5110P**
**Number of transistors**	2.3 billion	7.1 billion	5 billion
**Peak power consumption**	95 W	225 W	225 W
**Single precision floating point performance**	96 GFLOPS	3.5 TFLOPS	2.0 TFLOPS
**Main memory**	64 GB can be expanded	5 GB	8 GB

## Conclusion

We investigated how modern heterogeneous architectures cope with a selected computational problem typical for bioinformatics. The proof-of-concept implementation of SNPsyn on heterogeneous systems greatly reduces the (wall-clock) time needed for analysis of large GWAS data sets. GPUs proved to be a mature platform that offers a large amount of computing power to address inherently parallel problems, but is demanding for the programmer. A user who is only interested in using SNPsyn to analyze their data will profit the most by having multiple GPUs in their system. The new MIC architecture greatly alleviates programming but lacks in performance. Its ease of programming combined with good performance has a lot to offer to developers who don’t want to spend too much time optimizing their algorithms. Nevertheless, MIC is a general platform capable of tackling a wider range of more complex problems. This makes it very promising to excel in more complex analysis of SNP-SNP interactions such as adjustment for covariates [23].

## Availability and requirements

**Project name:** SNPsyn

**Project home page:**http://snpsyn.biolab.si

**Operating systems:** Linux, Windows, Mac OS

**Programming language:** C++

**Other requirements:** CUDA 2.0 or higher, Intel Composer XE 2013 or newer, make

**License:** GNU GPLv3

**Restrictions to use by non-academics:** none

## Competing interests

The authors declare that they have no competing interests.

## Authors’ contributions

UL, DS, TC, and BZ designed the study. DS implemented the CUDA and MIC software and measured the performance of the software. TC implemented the CPU and Python part of the software. DS wrote the first draft of the manuscript. All authors have written, read and approved the final manuscript.
